# The Design and Characterization of a Prototype Wideband Voltage Sensor Based on a Resistive Divider

**DOI:** 10.3390/s17112657

**Published:** 2017-11-17

**Authors:** Fernando Garnacho, Abderrahim Khamlichi, Jorge Rovira

**Affiliations:** Laboratorio Central Oficial de Electrotecnia (LCOE), Fundación para el Fomento de la Innovación Industrial (FFII), c/Eric Kandel, 1, 28906 Getafe, Madrid, Spain; ak@lcoe.etsii.upm.es (A.K.); jrovira@lcoe.etsii.upm.es (J.R.)

**Keywords:** resistive divider, voltage sensor, high-voltage calibration, electric field, frequency response, capacitive effect

## Abstract

The most important advantage of voltage dividers over traditional voltage transformers is that voltage dividers do not have an iron core with non-linear hysteresis characteristics. The voltage dividers have a linear behavior with respect to over-voltages and a flat frequency response larger frequency range. The weak point of a voltage divider is the influence of external high-voltage (HV) and earth parts in its vicinity. Electrical fields arising from high voltages in neighboring phases and from ground conductors and structures are one of their main sources for systematic measurement errors. This paper describes a shielding voltage divider for a 24 kV medium voltage network insulated in SF6 composed of two resistive-capacitive dividers, one integrated within the other, achieving a flat frequency response up to 10 kHz for ratio error and up to 5 kHz for phase displacement error. The metal shielding improves its immunity against electric and magnetic fields. The characterization performed on the built-in voltage sensor shows an accuracy class of 0.2 for a frequency range from 20 Hz to 5 kHz and a class of 0.5 for 1 Hz up to 20 Hz. A low temperature effect is also achieved for operation conditions of MV power grids.

## 1. Introduction

High-voltage (HV) dividers are a good alternative to traditional voltage transformers for power frequency measurements on medium- and high-voltage power lines [1,2]. An HV divider connected to a digital recorder is an appropriate measuring system for both continuous operation voltages (50 or 60 Hz, harmonics and subharmonics) and temporal or transient over-voltages (such as ferroresonances, switching, and lightning over-voltages) [3]. However, at present, the traceability for the measurement of harmonics and over-voltages is missing in most of the electrical grids because conventional voltage transformers are used.

Distributed generation of electric power, a common feature of smart grids, involves increasing levels of power electronics integration, such as power converters, electronic controllers, and loads with a greater number of semiconductor components. Consequently, the voltage wave of smart grids contains an increasing amount of harmonics. The international standards [4,5] evolved in recent years require measurements in frequency ranges up to 5 kHz. However, it is known that conventional measuring transformers [6,7,8,9] have a cut-off frequency no higher than 1 kHz. Furthermore, saturation phenomena appear for frequencies below grid frequency (50/60 Hz). For these reasons, new HV measuring systems have to be developed with metrological capabilities from a few hertz to several kilohertz for smart grids that are characterized in accordance with present international standards [10].

To date, high voltage resistive dividers [11,12] have not been used in electrical networks as voltage transfer devices. At first, this was due to the power supply requirements of voltmeters, energy meters and relays; at present, this is due to the different technical challenges to be solved, such as the capacitive influence of nearby metal parts connected to earth or high voltage that causes significant ratio and angle errors. Influence of overvoltages and high temperature changes are other challenges that must be solved. However, classical technology of HV dividers has been developed to design a double resistive-capacitive divider, one integrated inside the other, insulated in SF6, and with a flat frequency response (±0.2%) from 20 Hz to 5 kHz, for use in 20 kV power grids. The divider design and the electrical magnitudes of each component have been carefully chosen to achieve an industrial solution to be used in power grids. The prototype has passed the insulation coordination tests (125 kV for lightning impulses and 50 kV for power frequency voltage) maintaining its technical performances (a class of 0.2 from 20 Hz up to 5 kHz). This prototype opens a practical industrial approach to the on-line monitoring of power quality and to knowledge of grid overvoltages (temporary, switching, and lightning) to be supported by the grid components (power transformers, surge arresters, cables, etc.).

## 2. Voltage Sensor

### 2.1. Physical Design

**Figure 1:**
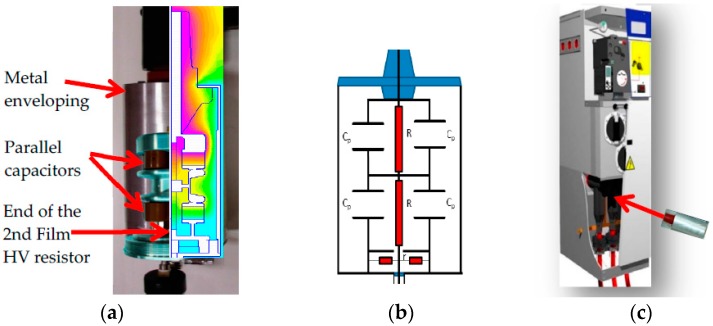
Voltage divider for MV switchgear under metal enclosure: (**a**) The cut-away view of the voltage divider enclosed in a metallic enveloping; (**b**) schematic electrical circuit; (**c**) general view of the HV divider sensor to be connected in a MV cabin.

**Figure 2:**
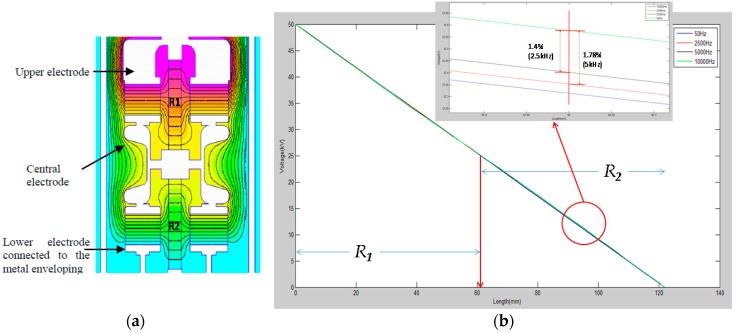
Voltage distribution: (**a**) Cut-away view of equipotential lines in the voltage divider; (**b**) voltage distribution along the two resistors of the HV branch for frequency range 50 Hz–5 kHz.

The voltage divider consists of a 50 MΩ HV resistive branch, composed of two HV film resistors, *R*_1_ and *R*_2_, of 25 MΩ, each one connected in series (Figure 1). The low-voltage (LV) resistive branch of the divider, *r*, of 50 kΩ is composed of four 200 kΩ resistors arranged in parallel in a coaxial configuration. Two blocks of four capacitors, with a rated capacitance of 202 pF each form two capacitances, *C_p_*, of 808 pF, connected in series The HV resistor is placed on the axis of the set of capacitors (Figure 1). The first block of capacitors is connected in parallel with the first resistor of the HV branch through an upper electrode and a central electrode. The second block of capacitors is connected between the central electrode and the metallic enclose (see Figure 1). The central electrode serves as mechanical support for the two capacitor blocks and the two resistors of the HV branch. The configuration is designed to achieve a voltage distribution along each HV resistor for higher frequencies as close as possible to the voltage distribution obtained for 50 Hz. The voltage distribution along the HV resistors was determined by FEM simulation for the frequency range from 50 Hz to 5 kHz (see Figure 2a). The central electrode is connected to joint point of both HV resistances. The set is in a steel–aluminum casing to achieve a good shielding. The LV branch is also arranged in an aluminum compartment, different to the HV branch, although sharing the same gas insulation. The SF6 gas at 0.2 MPa is used as an internal insulation to pass dielectric tests corresponding to the insulation level of 24 kV. A plug-in connector is used to be connected to the cable entry of the enclosed metal box.

### 2.2. Simplified Electrical Model

**Figure 3:**
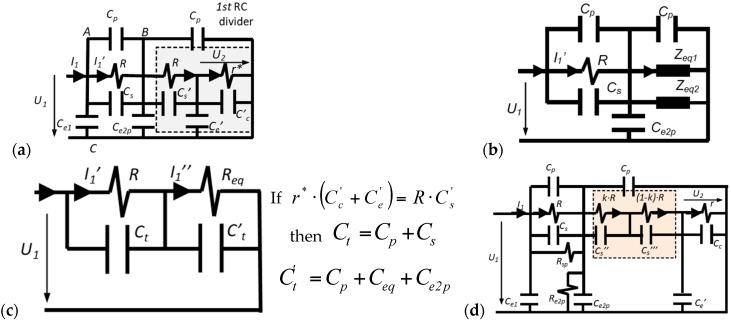
Equivalent electric schemes: (**a**) simplified divider model; (**b**) equivalent circuit replacing the 1st RC divider by two impedances in parallel *Z_eq_*_1_ and *Z_eq_*_2_; (**c**) equivalent model 2nd RC divider; (**d**) improved divider model analyzed in Section 4.

The simplified electric circuit of the double RC voltage divider is shown in Figure 3a. No inductance effect is considered because the cylindrical configuration of the divider and its size lead to an inductance less than 1 μH, which does not affect the frequency operation range of the divider. Each resistor of the HV branch, *R*_1_ and *R*_2_, is modeled through an ideal resistance, *R*, in parallel with a capacitance: *C_s_* for the first resistor *R*_1_ and *C_s_′* for the second one *R*_2_. The parallel capacitance *C_s_* and *C_s_′* includes not only the stray capacitance of the resistor but also the capacitance between the end electrodes of each HV resistor, *R*_1_ and *R*_2_. Consequently, a different value of these capacitances *C_s_* and *C_s_′* associated to each HV resistor is expected. The LV branch is represented by an ideal resistor *r* and a parallel capacitance *C_c_′* (see Figure 3a) in which two additional capacitive effects are included: the coaxial cable *C_c_* and of the digital recorder *C_r_* (20 pF). In practice, the impedance of the recorder is considered a resistance of 1 MΩ, r*, that must be added to the value of the LV resistance. The capacitances, *C_e_*_1_, *C_e_*_2*p*_, and *C_e_′*, represent the earth capacitive effect between the metallic enveloping and the upper electrode, the central electrode, and the LV resistor respectively. The *C_e_*_2*p*_ also includes any small difference between the first and the second capacitor blocks.

The circuit of Figure 3a is a simplified circuit for a specific frequency range to be determined. In this circuit, the LV branch (r*//*C_c_'* + *C_e_′*)) with the second block (*R//C_s_′*) of the HV branch form the first RC divider, whose equivalent circuit is composed of two parallel impedances *Z_eq_*_1_ and *Z_eq_*_2_ shown in Figure 3b.

(1) $$Z_{eq1}=\left(\frac{1-{\left(s\cdot c\right)}^{2}}{1-{\left(s\cdot a\right)}^{2}}\right)\cdot R+\left(\frac{1-{\left(s\cdot c\right)}^{2}}{1-{\left(s\cdot b\right)}^{2}}\right)\cdot r^{*}$$

(2) $$Z_{eq2}=\left(\frac{1-{\left(s\cdot c\right)}^{2}}{1-{\left(s\cdot a\right)}^{2}}\right)\cdot {\left(\frac{R}{R_{eq}}\right)}^{2}\cdot C_{s}^{\prime }+\left(\frac{1-{\left(s\cdot c\right)}^{2}}{1-{\left(s\cdot b\right)}^{2}}\right)\cdot {\left(\frac{r^{*}}{R_{eq}}\right)}^{2}\cdot \left(C_{c}^{\prime }+C_{e}^{\prime }\right)$$

where

(3) $$a=R\cdot C_{s}^{\prime },\;b=r^{*}\cdot \left(C_{c}^{\prime }+C_{e}^{\prime }\right),\;c=R_{eq}\cdot C_{eq}^{}$$

if the following condition is met:

(4) $$r^{*}\cdot \left(C_{c}^{\prime }+C_{e}^{\prime }\right)=R\cdot C_{s}^{\prime }$$

Thus, both impedances *Z_eq_*_1_ and *Z_eq_*_2_ become *R_eq_* and *C_eq_*:

(5) $$R_{eq1}=R+\cdot r^{*}$$

(6) $$C_{eq}={\left(\frac{R}{R_{eq}}\right)}^{2}\cdot C_{s}^{\prime }+{\left(\frac{r^{*}}{R_{eq}}\right)}^{2}\cdot \left(C_{c}^{\prime }+C_{e}^{\prime }\right)$$

Consequently, the circuit of Figure 3b leads to the circuit of Figure 3c in the second RC divider, in which the first RC divider is therein. It justifies the name “double RC divider” that the authors have given to this voltage sensor. Using this design, the earth capacitances *C_e_*_2_ and *C_e_′* became a part of the total parallel capacitances of the circuit shown in Figure 3c. An appropriate selection of the capacities *C_e_*_2*p*_, *C_e_′*, *C_s_*, *C_s_′*, and *C_c_* is required to compensate the ratio and phase displacement errors of the divider. An improved model is shown in Figure 3d, in which the main difference from the simplified one is that the second resistor *R* of the HV branch is split into two parts and a stray capacitance is associated with each one. In addition, a parallel leakage resistance is introduced in each capacitor block to represent its insulation resistance. The behavior of this improved model is explained in detail by simulation in Section 4.

The transfer function of the divider is given by the following formula:

(7) $$G_{d}^{}(s)=\frac{U_{2}}{U_{1}}=r\cdot \frac{1+s\cdot R_{eq}\cdot C_{eq}^{}}{1+s\cdot r^{*}\cdot C_{c}^{\prime \prime }}\cdot \frac{1}{\left[R_{eq}+R\cdot \left(\frac{1+s\cdot R_{eq}\cdot C_{t}^{\prime }}{1+s\cdot R\cdot C_{t}^{}}\right)\right]}.$$

For direct voltage (*s* = 0) the transfer function is transformed in the following:

(8) $$G_{d}(s=0)=\frac{r^{*}}{R_{eq}+R}$$

and the normalized Laplace transfer function (G*_nd_ (*s* = 0) = 1) by the following one:

(9) $$G_{nd}^{*}(s)=\frac{G_{d}(s)}{G_{0}(s=0)}=\frac{R+R_{eq}}{R_{eq}}\cdot \frac{1+s\cdot R_{eq}\cdot C_{eq}}{1+s\cdot r^{*}\cdot C_{c}^{\prime }}\cdot \frac{1}{\left[1+\frac{R}{R_{eq}}\cdot \left(\frac{1+s\cdot R_{eq}\cdot C_{t}^{\prime }}{1+s\cdot R\cdot C_{t}}\right)\right]}.$$

Each RC divider should be designed to meet the following requirements:

Requirement of the 1st RC divider:

(10) $$R\cdot C_{s}^{\prime }=r^{*}\cdot (C_{c}^{\prime }+C_{e}^{\prime })=r^{*}\cdot C_{c}^{\prime \prime }$$

Requirement of the 2nd RC divider:

(11) $$R_{eq}\cdot C_{t}^{\prime }=R\cdot C_{t}^{}$$

where

(12) $$C_{t}^{}=C_{p}^{}+C_{s}^{},\;C_{t}^{\prime }=C_{p}+C_{eq}^{}+C_{e2p}$$

If both RC dividers meet these requirements, the normalized transfer function will be 1 p.u. The capacitances, *C_s_* and *C_s_′*, must be designed to comply with Equation (13), taking into account Equation (6):

(13) $$C_{s}=\frac{R_{eq}}{R}\cdot C_{eq}+\frac{R_{eq}}{R}\cdot C_{e}+\frac{\left(R_{eq}-R\right)}{R}\cdot C_{p}$$

and the length of the coaxial cable must be chosen to comply the following condition:

(14) $$\mathrm{Length}\;\mathrm{coaxial}\;\mathrm{cable}=\frac{C_{c}^{\prime }-C_{r}}{c_{c}}$$

where *c_c_* is the capacitance per length unit of the coaxial cable of 66 pF/m, and *C_r_* is the capacitance of the digital recorder.

## 3. Frequency Response Analysis

### 3.1. Frequency Response Analysis Using the Simplified Model

In general, Equations (10) and (11) are not fully complied. One of the most common causes is the cable length of the coaxial cable used to connect with the digital recorder that modifies the *C_c_* value. It can be slightly different from the theoretical value that satisfies Equations (10) and (11). The theoretical ratio error and the phase displacement error caused by the length of the coaxial cable can be determined using the transfer function, i.e., Equation (9), derived from the simplified circuit. In this simplified circuit, the theoretical maximum ratio error caused by the cable length of the coaxial cable can be determined through that equation for *s* → ∞:

(15) $$\lim_{s\to \infty }G_{nd}(s)=\frac{R_{eq}+R}{r^{*}}\cdot \frac{C_{eq}^{}}{C_{c}^{\prime }}\cdot \frac{C_{t}^{}}{\left(C_{t}^{}+C_{t}^{\prime }\right)}$$

To apply Equation (9) to the designed sensor, its electrical parameters must be determined. Most of them are determined by measurements. The electrical data of the digital recorder (*Z_r_* and *C_r_*) are collected from the manufacturer’s data sheet. Other parameters were estimated by modeling in order to achieve the best fitting to the actual measured in the laboratory. All parameter values of the built HV sensor are shown in Table 1.

**Figure 4:**
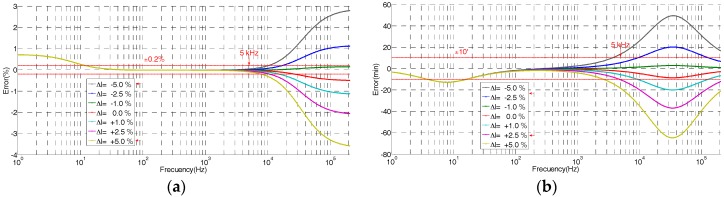
Errors due to difference of length of coaxial cable *C_c_* regarding to the theoretical value regarding the simplified model of Figure 3: (**a**) ratio error; (**b**) phase displacement error.

The theoretical frequency response derived from Equation (9) of the built HV sensor is shown in Figure 4 for different coaxial cable lengths. The difference between the real length of the coaxial cable and the reference length value given by Equation (10) provokes a clear ratio and angle errors for frequencies higher than 2 kHz. When the cable length is larger than the reference value, a negative ratio error is expected (see Figure 4). Length variations around ±5% (around ±5 cm of the coaxial cable) are acceptably to keep ratio error within an accuracy class of 0.2, but length variations lower than ±2.5% are recommended if the phase displacement error is to be kept within the admissible limits for a maximum frequency of 5 kHz. This length requirement for the coaxial cable can be easily fulfilled if the global measuring set, composed of the HV divider, a coaxial cable, and a digital recorder, is supplied by the same manufacturer.

**Figure 5:**
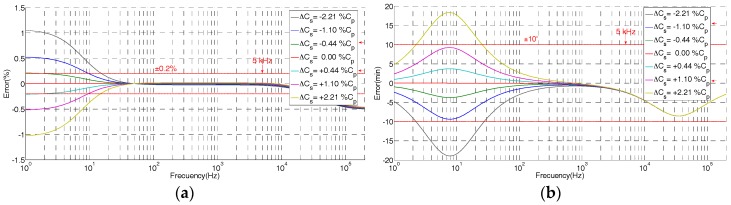
Errors due to different *C_s_* values using the simplified model of Figure 3: (**a**) ratio error; (**b**) phase displacement error.

The theoretical frequency response derived from the simplified model (Equation (9)) is shown in Figure 5 for different *C_s_* values referred to a percentage of *C_p_*. It justifies the change of the ratio error and angle error for lower frequencies due to changes of the *C_s_* value regarding the reference value given by Equation (11).

### 3.2. Measuring the Response Frequency of the Built Sensor

Taking into account the data of Table 1, the two restrictions referred in Equations (10) and (11) are checked for the built sensor (see Table 2). It can be observed that Equation (10), corresponding to the restriction of the 1st divider, is better complied (0.5%) with than the restriction given by Equation (11) associated with the 2nd divider (1.5%). The deviation due to the 2nd divider is caused by a real value of *C_s_* = 2.2 pF (measured) when it should be 14.3 pF to satisfy Equation (11). An improved design of the upper electrode and the central electrode would permit a reduction in this discrepancy in order to obtain a class of 0.2 from 1 Hz to 5 kHz with the same divider ratio value, as is shown in Figure 5a.

**Figure 6:**
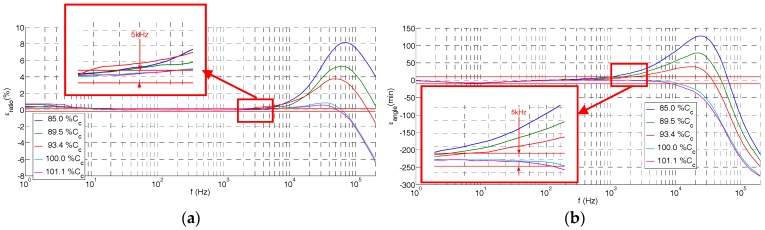
Measured errors in the real design of Figure 1 due to the difference in the length of coaxial cable *C_c_* with respect to the theoretical value: (**a**) ratio error; (**b**) phase displacement error.

The frequency response of the built voltage sensor was measured (see Figure 6) for different *C_c_* values (different cable lengths) in the LCOE calibration laboratory in order to check Equation (9) of the simplified model. It was also observed that, for lengths of the coaxial cable larger than the reference value (97 cm), the tendency of the ratio error and the angle error is negative for higher frequencies (>200 kHz) according to the simplified model, but a preliminary oscillation is observed in the interest frequency range (2–100 kHz). This effect is not detectable by the simplified model. For this reason, the improved model shown in Figure 3d is introduced in Section 4.

**Figure 7:**
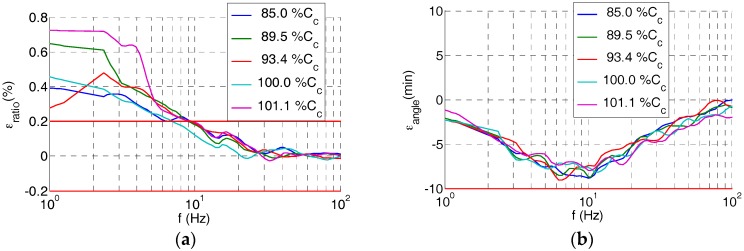
Measured errors of the built HV sensor in the low frequency range for difference lengths of the coaxial cable *C_c_*: (**a**) ratio error; (**b**) phase displacement error.

In the low frequency range (1–10 Hz), the rated ratio of the divider increases up to 0.7% (see Figure 7), while the angle error maintains lower than 10 min for the *C_c_* values analyzed (85.0–101.1% *C_c_*).

**Figure 8:**
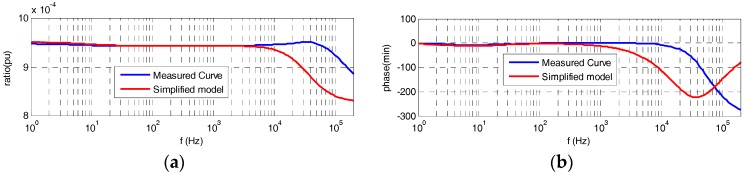
Frequency response curves: the red curve is obtained via Equation (9) and the blue curve was measured in the LCOE laboratory: (**a**) ratio error; (**b**) phase displacement error.

Both frequency responses, of the simplified model derived from Equation (9) and of the built divider measured in the LCOE laboratory, are shown in Figure 8. It can be observed that the simplified model follows the real frequency behavior for a frequency range up to 3 kHz.

## 4. Improved Electrical Model

**Figure 9:**
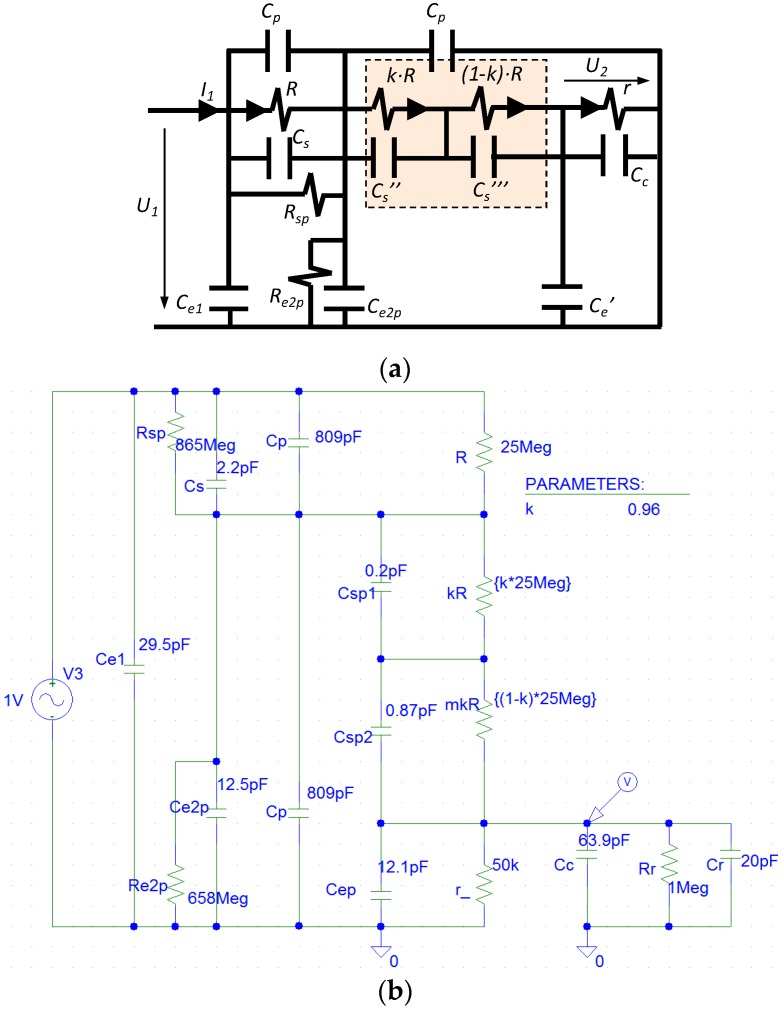
Improved model of the sensor: (**a**) equivalent electric circuit; (**b**) PSPICE model to fit the frequency response to the measured one.

**Figure 10:**
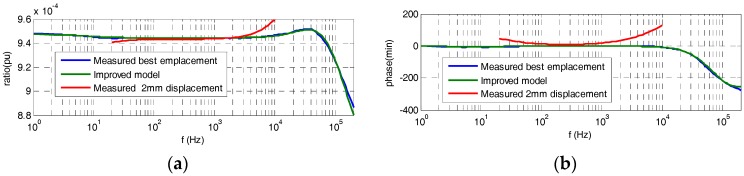
Frequency response curves measured with the best emplacement of the 2nd HV resistance (blue curve), with a vertical displacement of 2 mm (red curve), and obtained by PSPICE simulating the frequency response of best emplacement of the 2nd HV resistance (green curve) using the coefficient *k* = 0.96 to the frequency response measured: (**a**) ratio error; (**b**) phase displacement error.

To improve the simplified model shown in Figure 3a, the 2nd resistor of the HV branch is split into two parts, as is shown in the circuit of Figure 3d. Each resistance part *k·R* and (1 *− k*) *· R* has a different stray capacitance *C_s_′′* and *C_s_′′′* in parallel depending on the geometrical location between the resistance and the central electrode (see Figure 2a), which it is considered in the PSPICE circuit shown in Figure 9 by *C_sp_*_1_ (*C_s_′′*) and *C_sp_*_2_ (*C_s_′′′*), respectively. For the built sensor, an equivalent capacitance of *C_s_′′* is in parallel with the first part of the resistance part *k·R* and another capacitance of *C_s_′′′* is in parallel with the other resistance part (1 *− k*) *· R*. The values of the coefficient *k* (0.96) and of the capacitances *C_s_′′* (0.20 pF) and *C_s_′′′* (0.87 pF) have been determined by an iterative process by means of circuit analysis and synthesis using PSPICE modeling and MATLAB in order to fit the theoretical curve of the frequency response to the real one measured in the laboratory. Furthermore, two additional resistors (*R_sp_* and *R_e_*_2*p*_) that mainly represent leakage resistances of both capacitor blocks are also added to simulate in a better way the real behavior of the built divider. The *R_sp_* magnitude also includes any difference between the 1st and the 2nd HV resistances. The improved model achieves a good fitting to the measured frequency response (see Figure 10) for the set values of the parameters of Table 1, while the simplified model does not fit the high frequency range (Figure 8). The frequency response measured in the built divider with a small change in the emplacement of the 2nd resistance is also included in Figure 10. The emplacement change moved vertically 2 mm from the relative position of the 2nd resistance regarding the central electrode. A significant influence in the frequency response curve can be observed, which justifies the inclusion of stray capacitances *C_s_′′* and *C_s_′′′* in the improved circuit model shown in Figure 3d and in its simulate PSPICE circuit shown in Figure 9.

## 5. High-Voltage Calibration and Insulation Testing

### 5.1. Ratio and Angle Errors

**Figure 11:**
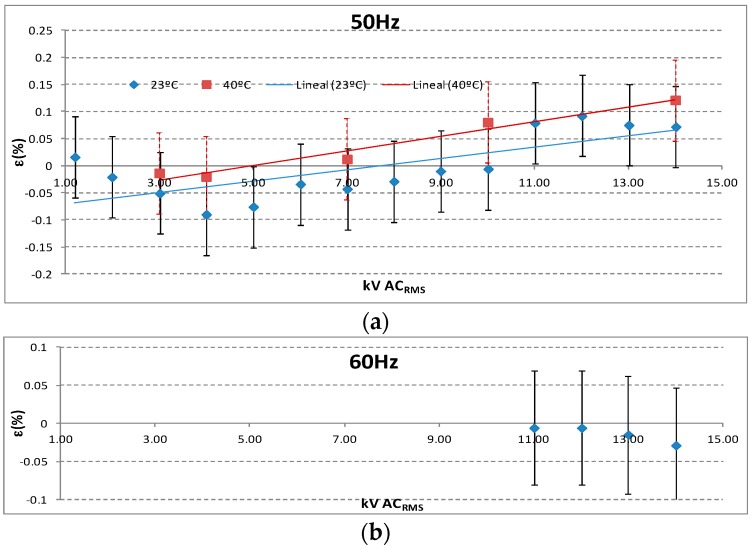
(**a**) Ratio errors for HV calibration from 1 to 14 kV, 50 Hz at 20 °C and 40 °C, (**b**) ratio errors for HV calibration from 11 to 14 kV, 60 Hz at 20 °C.

The voltage sensor was calibrated in a HV range from 1 to 14 kV (24/√3 kV) for a sinusoidal frequency of 50 Hz using voltage standard transformers. The calibration was complemented with a power frequency test of 60 Hz at 14 kV (24/√3 kV). An uncertainty of 0.08% for the HV calibration was achieved. The calibration was performed for two environment temperatures: 20 °C and 40 °C. The ratio errors obtained are inside the admissible tolerances corresponding to an accuracy class of 0.2 (see Figure 11).

### 5.2. Insulation Tests

**Figure 12:**
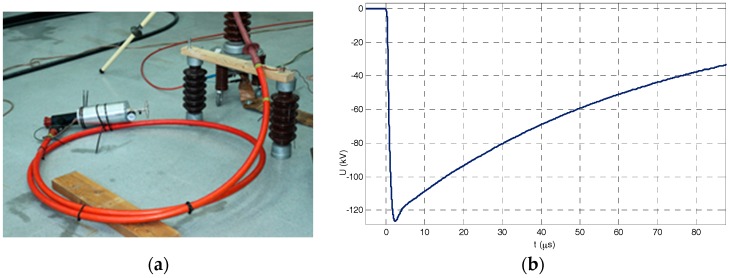
(**a**) Testing layout of the voltage divider prototype connected to a medium voltage cable, (**b**) negative lightning impulse (125 kV) applied during the withstand test.

Insulation tests were usefully passed corresponding to the insulation level for material to be used in a power grid of 24 kV. Fifteen positive and negative lightning impulses 1.2/50 of 125 kV were applied without any breakdown and a power frequency voltage of 50 kV (*U_peak_*/√2) for a minute was applied without any breakdown (see Figure 12).

## 6. Conclusions

A voltage sensor on the basis of a shielding double RC voltage divider has been designed, developed, built, and tested. The design is based in two RC dividers, one inside the other. This design permits to transform earth capacitances of the HV resistive branch to parallel capacitances. The frequency response of the built sensor shows a flat frequency response from 20 Hz to 5 kHz, with a ratio error and a phase displacement error inside the admissible errors of a class of 0.2. The class increases to 0.5 if the sensor is used from 1 to 20 Hz. An optimized design to maintain the 0.2 class from DC to 5 kHz is attainable if the parallel capacitance *C_s_*, of the first HV resistor, *R*, is increased. The insulation tests demonstrate that the built sensor can be used in power grids up to 24 kV.

## Figures and Tables

**Table 1:** Electrical parameters of the built high-voltage (HV) sensor.

Parameter	Primary Value	Determined By	Simplified Model Figure 3b	Simplified Model Figure 3b	Improved Model Figure 3d
*R*	25 MΩ	Measurements	25 MΩ	25 MΩ	25 MΩ
*r*	50 kΩ	Measurements	-	-	-
*Z_r_*	1 MΩ	Data Sheet	-	-	-
r* = r·Z_r_/(r + Z_r_)	-	Derived	47.6 kΩ	47.6 kΩ	47.6 kΩ
*C_p_*	809.2 pF	Measurements	809.2 pF	809.2 pF	809.2 pF
*C_c_* ^(1)^	63.8 pF	Measurements	-	-	-
*C_r_*	20.0 pF	Data sheet	-	-	-
*C_c_′ = C_c_ + C_r_*	83.8 pF	Derived	-	-	-
*C_e_′*	12.1 pF	Measurements	12.1 pF	12.1 pF	12.1 pF
*C_c_′′ = C_c_′+ C_e_′*	-	Derived	95.9 pF	95.9 pF	95.9 pF
*C_e_*_1_^(3)^	29.5 pF	Measurements	-	-	29.5 pF
*C_e_*_2*p*_	12.5 pF	Measurements	12.5 pF	12.5 pF	12.5 pF
*C_s_*	2.2 pF	Measurements	2.2 pF ^(2)^	2.2 pF ^(2)^	2.2 pF ^(2)^
*C_s_′*	0.18 pF	Modeling	0.18 pF	0.18 pF	-
*C_s_′′/C_s_′′′*	-	Modeling	-	-	0.20/0.87 pF
*R_equ_*	-	Derived (5)	25 048 kΩ	25 048 kΩ	-
*C_equ_*	-	Derived (6)	0.15 pF	-	-
*C_t_*	-	Derived (12)		811.4 pF	-
*C_t’_*	-	Derived (12)	-	821.9 pF	-
*R_s_*	-	Modeling	-	-	865 MΩ
*R_e_*_2_	-	Modeling	-	-	658 MΩ
*k*	-	Modeling	-	-	0.96

^(1)^ This capacitance corresponds to a length of the coaxial cable 97 cm (66 pF/m). ^(2)^ The measured value is different to the reference value 14.3 pF given by Equations (11) and (12). ^(3)^ The capacitance difference between the magnitudes of the 2nd capacitor block *C_p_* and the 1st one is included in the *C_e_*_2*p*_ value.

**Table 2:** Checking restrictions of Equations (10) and (11) for the built voltage sensor.

Restriction of 1st RC Divisor	Equation (1) *R*·*C_s_′* = r*·(*C_c_′* + *C_e_′*)	Restriction of 2nd RC Divisor	*R·C_t_* = *R_eq_·C_t_′*
*R·C_s_′* (kΩ*·*pF)	4545	*R·C_t_* (kΩ*·*pF)	20,284
r*·(*C_c_'* + *C_e_′*) (kΩ*·*pF)	4568	*R_eq_·C_t_'* (kΩ*·*pF)	20,587
